# What do we know about the non-work determinants of workers' mental health? A systematic review of longitudinal studies

**DOI:** 10.1186/1471-2458-11-439

**Published:** 2011-06-06

**Authors:** Nancy Beauregard, Alain Marchand, Marie-Eve Blanc

**Affiliations:** 1 School of Industrial Relations, University of Montreal, P.O. Box 6128, Downtown Station, Montreal, H3C 3J7, Quebec, Canada; 2 University of Montreal Research Institute in Public Health, Montreal, Quebec, Canada

## Abstract

**Background:**

In the past years, cumulative evidence has convincingly demonstrated that the work environment is a critical determinant of workers' mental health. Nevertheless, much less attention has been dedicated towards understanding the pathways through which other pivotal life environments might also concomitantly intervene, along with the work environment, to bring about mental health outcomes in the workforce. The aim of this study consisted in conducting a systematic review examining the relative contribution of non-work determinants to the prediction of workers' mental health in order to bridge that gap in knowledge.

**Methods:**

We searched electronic databases and bibliographies up to 2008 for observational longitudinal studies jointly investigating work and non-work determinants of workers' mental health. A narrative synthesis (MOOSE) was performed to synthesize data and provide an assessment of study conceptual and methodological quality.

**Results:**

Thirteen studies were selected for evaluation. Seven of these were of relatively high methodological quality. Assessment of study conceptual quality yielded modest analytical breadth and depth in the ways studies conceptualized the non-work domain as defined by family, network and community/society-level indicators. We found evidence of moderate strength supporting a causal association between social support from the networks and workers' mental health, but insufficient evidence of specific indicator involvement for other analytical levels considered (i.e., family, community/society).

**Conclusions:**

Largely underinvestigated, non-work determinants are important to the prediction of workers' mental health. More longitudinal studies concomitantly investigating work and non-work determinants of workers' mental health are warranted to better inform healthy workplace research, intervention, and policy.

## Background

For the past three decades, epidemiological research, influenced predominantly by the Demand-Control-Support[1] and Effort-Reward Imbalance[2] models, has highlighted the connection between key features of the psychosocial work environment (e.g., decision latitude, psychological demands, social support, rewards) and the deterioration of workers' mental health. This substantial body of work has recently been the focus of several systematic reviews of work-specific determinants[3-5] and leveraged interventions[6-10]. Interestingly, the literature has devoted much less attention to understanding the pathways through which other pivotal life environments might also concomitantly intervene, along with the work environment, to bring about improved mental health outcomes in the working population[11-13]. The current study seeks to bridge this gap by systematically reviewing the relative contribution of non-work determinants to workers' mental health[14].

### The non-work domain: Conceptual and analytical considerations

The construct "non-work domain" has taken on multiple meanings in the literature on workers' mental health, ranging from chronic stressors and life events[15] to the inclusion of health-related lifestyles and symptoms[16]. Such broad conceptual heterogeneity in the non-work domain construct represents a significant limitation for advancing research on workers' mental health since the specific contribution of non-work determinants remains diffuse and unclear.

In the interest of conceptual clarity, we have borrowed from past work on social structures, agency and workers' mental health to delineate specific constitutive attributes of the non-work domain [12,13]. In line with the sociological theory of agency-structure [17,18], we view macro- (e.g., society), meso- (e.g., workplace, networks) and microsocial structures of the daily life (e.g., family) as many life environments in which workers routinely find themselves.

Following from this, workers' mental health can be conceptualized as resulting from the cumulative opportunity structures and constraints embedded in these life environments to which workers are exposed [12,13,19]. Consequently, workers' mental health becomes not only rooted in the work environment, but also in other pivotal life environments such as the family, networks, community, and, more broadly, the society to which workers belong. These other life environments constitute what we define here as the non-work domain. The attributes of the non-work domain are thus of inherently social nature, and should analytically be distinguished from any specific attributes pertaining to the workers as individual agents encompassing notably "reflectiveness, rationality, creativity, demography, affect, the body, biology, representations, perceptions, motivations, habits, and attitudes" [12].

This systemic approach of the non-work domain is congruent with integrative work on social integration [20] and on psychosocial risk factors of home and community settings [21]. Accordingly, the non-work domain can be posited to shape workers' mental health through causally and dynamically intertwined mechanisms at three levels of analysis: 1) the macrosocial level of community or society (e.g., culture, socioeconomic factors); 2) the mesosocial level of networks (e.g., social network structures, characteristics of network ties); and 3) the microsocial level of the family unit (e.g., marital and parental relationships).

Furthermore, in line with recent studies on the material and psychosocial pathways of health[22,23], we have posited that each non-work analytical level and its constitutive mechanisms are distinctly linked to workers' mental health outcomes through objective and subjective measures of non-work determinants.

Based on the propositions mentioned above that define the non-work domain construct, this systematic review aims to answer the following research question: What is the nature of the causal association between non-work determinants and workers' mental health, once the concomitant contribution of work determinants is accounted for?

## Methods

### Definition and inclusion parameters

This systematic review examined the concomitant causal association between work and non-work determinants of workers' mental health. The definition of mental health put forth encompassed three widely investigated outcomes: psychological distress, depression, and burnout. Work exposure referred to the psychosocial work environment described by the Demand-Control-Support model[1], the Effort-Reward model[2], and any related concepts (e.g., organizational justice), as well as objective features of the work contract (e.g., working hours). Drawing from our framework, we defined non-work exposure from the levels of analysis (e.g., family, networks, community, society) that describe the non-work domain[12,20].

Eligibility criteria for selecting the studies that best captured the nature of the explanatory dynamics investigated focused on observational longitudinal studies of working-age adults. In order to minimize bias, the study design specified the following inclusion parameters. Firstly, we opted for community-based as opposed to clinical-based sampling of workers to ensure that selected workers were not followed for other concurrent medical conditions implicating potential reverse causation effects of mental health on workers' assessment of their work and non-work exposures[24]. Secondly, a sample size of at least 200 workers was chosen in order to make reasonable statistical power assumptions about the investigated work and non-work exposures. With a conservative variable-to-cases ratio of 1:10 [25], we estimated that comprehensive studies based on extensive work exposure (e.g., indicators from the Job-Demand-Control and Effort-Reward Imbalance models), non-work exposure (i.e., family, network and community/society-level indicators) and adjustment strategies (e.g., lifestyles, sociodemographic profile, chronic health conditions) would be optimally targeted by our research question. Thirdly, a minimum observation period of at least 12 months in order for work exposure to have a stable effect on mental health was also observed in conformity with similar research efforts[5]. Fourthly, a multivariate evaluation of work and non-work exposures with reports of their respective effect sizes was required. Measurements for mental health also needed to be based on multidimensional, psychometrically sound instruments; therefore, we considered both continuous and binary statistical treatments of mental health outcomes[26,27]. Lastly, this review focused on empirical research published in English and French (grey literature excluded).

### Search strategy

A comprehensive search strategy was designed to assessed the non-work domain in the literature [28]. Multiple databases were queried from the start date through July 26, 2008: Cinhal (Ovid), Psycinfo (Ovid), Embase (Ovid), Medline (Ovid), EBM (all databases, Ovid), Sociological Abstracts (ISI Web), the Social Sciences Citation Index (ISI Web), and the Arts and Human Citation Index (ISI Web). We elaborated an electronic search strategy that included indexed and free terms in keeping with similar research on outcomes[5,7] and work-exposure definitions[3,5], although we deduced non-work exposure from our framework (see Additional file 1). We also conducted a confirmatory search strategy based on an inductive screening of bibliographies from potentially eligible studies, relevant reviews[3-5,24], and an electronic search of Medline (OVID) from 2005 to 2008 that omitted the non-work exposure filters introduced in the original search strategy.

### Data extraction and management

We applied a two-stage selection process to data extraction. In the first stage, we examined titles and abstracts to ascertain potential study eligibility. One researcher conducted the first-stage iteration, which a second researcher then corroborated using a random subsample (*N *= 240, kappa = 0.89). One researcher conducted the second data-extraction stage, which focused on full-text, potentially eligible studies. Disagreements throughout both the extraction and appraisal phases were resolved by discussion with a third researcher. We used Nvivo 2.0 and SPSS 15.0 to manage data from the extraction phase[29,30].

### Critical appraisal and synthesis of the evidence

As anticipated, the heterogeneity in the conceptualization and operationalization of the non-work domain precluded meta-analysis of the data. We therefore opted for a narrative synthesis based on a critical appraisal that included both conceptual and methodological considerations (see Additional file 2). The conceptual component of the critical appraisal examined the level of comprehensiveness associated with the conceptualization and operationalization of the non-work domain and comprised two components.

*Analytical breadth *corresponded to the number of analytical levels (e.g., family, networks, community, society) considered by the included studies. For clarification purposes, we attributed analytical levels as follows: 1) the family modality referred specifically to workers' partner, children, and parents; 2) the network modality referred to relatives, friends, and generic references to social relationships (e.g., "people"); and 3) the community/society modality referred to community or societal features (e.g., occupational status based on national classification systems). Scores were derived additively if more than one analytical level was present for a given study, higher scores indicating greater analytical breadth. Illustratively, joint inclusion of family and community levels in single or multiple indicators would earn two stars out of a possibility of three stars.

*Analytical depth *measured the extent to which, for a given analytical level, multiple indicators of the non-work domain were included within studies. We distinguished among low (1 indicator), moderate (2 or more indicators), and high (2 or more indicators of a single construct with both objective and subjective assessments) levels of analytical depth. Objective indicators comprised social position markers (e.g., marital status) or cumulative exposure to non-work factors (e.g., life events)[31], whereas subjective indicators comprised workers' appraisals of the level of stressfulness experienced relative to non-work factors.

Once we had mapped conceptual comprehensiveness, we measured *methodological quality *using the Newcastle Ottawa Scale (NOS)[32,33], a validated 9-item questionnaire that evaluates design robustness based on cohort selection, adjustments for confounding factors, and ascertainment of exposures or outcomes. We adopted a descriptive approach to characterize the strength of the evidence, using a multiple-criteria triangulation[34]. Three criteria were examined: 1) adequacy of methodological quality (relatively high methodological quality set at NOS score > mean NOS score); 2) consistency of findings (at least 75% of the studies reporting a significant finding at *p *< 0.05 in the anticipated direction for exposure-outcome association); and 3) strength of causal association (strong magnitude set at OR≥2.0 or ≤0.75). We considered strength of evidence "strong" if all three criteria were cumulatively satisfied (i.e., at least 75% of relatively high-quality studies reporting results of strong magnitude), "moderate" if consistent results were obtained from high-quality studies only or a mixture of high- and low-quality studies in the anticipated direction for exposure-outcome association independently from the strength of association, and "insufficient" if consistency could not be reached or was based on low-quality studies only (see Additional file 3 for further details on the decision process followed). In determining the strength of evidence, we duplicated observations at each analytical level considered for such cases where analytical levels could not specifically be untangled from indicator measurement.

Because all studies provided direct evidence or references with sufficient information for valid evaluations to be made, it was not necessary to contact any study authors for clarification. In order to minimize potential conflation bias in the results, studies from a single cohort sharing partial data overlap were examined separately provided that they cumulatively present: a) different endpoints; and b) a combination of substantively different work and non-work exposures, and mental health outcomes. In the presence of multiple non-independent studies and studies relying on multiple analytical strategies, we selected the study yielding the highest NOS score. Two reviewers independently performed iterations in the critical appraisal phase, with interrater agreement levels for methodological and conceptual components estimated at kappa = 0.79 and kappa = 0.76 respectively. This systematic review follows the recommendations of the MOOSE guidelines (see Additional file 4) [14].

## Results

**Figure 1:**
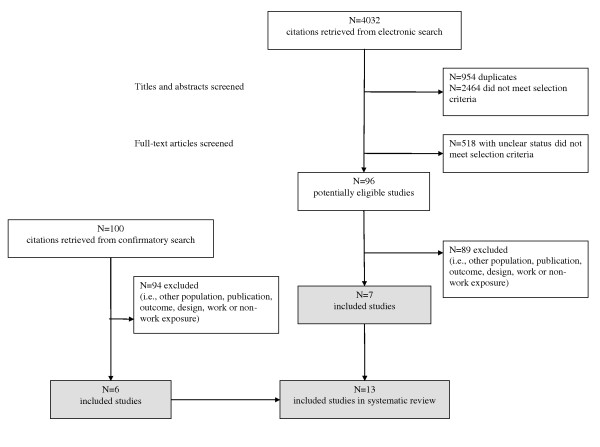
**Flow diagram of cross-sectional identification and retrieval of examined studies**.

We retrieved a total of 4,032 studies from the original literature search. Of the 96 studies identified as potentially eligible, we selected 7 for review[12,35-40]. Reasons for exclusions were: cross-sectional design[41,42], sample size[43-48], observation period[49-56], lack of conformity with outcome[57-59], or work exposure definition[60-76]. From those studies meeting the preceding criteria, we excluded additional studies based on the absence of non-work exposure[77-103], failure to report size effects for non-work exposure[104-126], univariate examination of work and non-work factors[127], and non-independent samples[13,46,128]. We retrieved six additional studies from the confirmatory search (full details available from authors)[129-134]. Figure 1 illustrates the selection process followed to determine eligibility. Included studies comprised 12 prospective cohort studies[12,36-40,129-134] and 1 retrospective case-control study[35].

Table 1 summarized the characteristics of the included studies. All studies were conducted in Europe or North America. Among the 13 studies reviewed, 6 were derived from independently designed longitudinal cohorts [35,36,38-40,129], while the 7 remaining studies involved two independently designed longitudinal cohorts, namely the National Population Health Survey [12,130,132,133], and the Whitehall study [37,131,134]. A wide range of outcome measurement was used for psychological distress or depression, burnout was not investigated by any studies. The follow-up period varied from 1 to 10 years. All studies controlled for sociodemographic profile (e.g., age, gender), with fewer reports of adjustments for past mental health history, personality traits and lifestyles.

**Table 1:** Description of included longitudinal cohort and case-control studies

References	Sample	Mental health	Follow-up (years)	Non-work factors	Adjustments
**Cohorts**					
Barnett and Brennan (1998) [40]	Full-time employed, dual- earner couples (*N *= 484). United States.	Psychological distress (SCL-90)	2	*Community* Occupational prestige: men = ns, women = ns *Family* Years in couple: men and women b = -0.22/ Marital-role quality: men and women b = -2.71/ Household income: men = ns, women = ns/ Children at home: men = ns, women = ns	Age, gender as a stratification variable, education, negative affectivity, partners' psychosocial work environment, mental health at baseline, skill discretion, decision authority, schedule control, job demands, pay adequacy, job security, social support, work hours.

Bromet et al. (1988) [39]	Married male power plants employees (*N *= 325). United States.	Depression (SADS-L) Psychological distress (SCL-90)	1	*Networks* Social support (friends) = ns *Family* Marital stress = ns	Age, history of affective disorders, levels of psychological distress at baseline, alcohol-related problems, decision latitude, job demands, social support at work.

Fuhrer et al. (1999) [131]	Civil servants aged 35-55 years at baseline (*N *= 5,793). United Kingdom.	Psychological distress (GHQ)	Up to 4	*Combined Community and Networks* Social network index (friends, relatives; church, social clubs): men = ns, women = ns *Combined Networks and Family* Confiding support: men OR_low _= 1.24, women = ns/Practical support: men = ns, women = ns/Negative aspects of close relationships: men OR _moderate, high _= 1.41-1.80, women OR_moderate, high _= 1.39-2.06 (all close nominated persons in reference for all indicators, with spouse in reference for 80-92% of respondents) *Family* Marital status: men = ns, women = ns.	Age, gender as a stratification variable, employment grade, mental health at baseline, social support at work.

Griffin et al. (2002) [37]	Civil servants aged 35-55 years at baseline (*N *= 7,473). United Kingdom.	Depression (GHQ) Anxiety (GHQ) Exclusion of cases at baseline.	5	Depression *Networks* Caregiving status (relative): men OR = 1.59, women = ns *Family* Marital status: men = ns, women = ns/Number of children: men = ns, women = ns/Home control: men OR_low _= 1.71, women OR_low _= 2.02 Anxiety *Networks* Caregiving status (relative): men OR = 1.70, women = ns *Family* Marital status: men = ns, women = ns/Number of children: men = ns, women = ns/Home control: men OR_low _= 1.68, women = ns	Age, gender as a stratification variable, employment grade, decision latitude.

Marchand et al. (2005)[12]	Representative sample of the workforce (*N *= 6,359). Canada.	Psychological distress (WHO-CIDI)	7	*Community* Societal occupational structure accounts for 1.3% of variance in outcome/Mean occupational income = ns *Networks* Social support (someone): OR_high _= 0.58 *Family* Marital status = ns/Couple strains = ns/Household income = ns/Children aged 0-5 yo at home = ns/Children aged 6-11 yo at home = ns/Children aged 12-24 yo at home = ns/Children strains OR = 1.15	Age, gender, self-esteem, locus of control, sense of cohesion, chronic health problems, alcohol consumption, smoking, physical activity, stressful childhood events, mental health at baseline, skill utilization, decision authority, job demands, physical demands, social support at work, job insecurity, work hours, work schedule.

Niedhammer et al. (1998)[38]	Workers from public utility energy firms aged 35-50 years at baseline (*N *= 9,059). France.	Depressive symptoms (CES-D)	1	*Combined Networks and Family* Nb. of life events (partner; relatives): men OR = 1.15-1.77, women OR = 1.53-3.17 *Family* Marital status: men OR_single, separated, divorced, widowed _= 1.72-2.88, women OR_separated, divorced, widowed _= 1.36-2.16	Age, gender as a stratification variable, education, occupational status, stressful occupational events, previous absenteeism for mental health, decision latitude, job demands, social support at work.

Revicki et al. (1993) [129]	Emergency medicine residents (*N *= 369). United States.	Depression (CES-D)	1	*Family* Marital status = ns	Age, gender, mental health at baseline, Work-Related Stress Inventory, task-role clarity, social support at work.

Shields (1999) [132]	Workers aged 25- 54 years working a minimum of 35 hours per week (*N *= 3,783). Canada.	Major depressive episodes (WHO-CIDI) Exclusion of cases at baseline.	2	*Community* Occupational status: men = ns, women = ns *Family* Marital status: men = ns, women = ns/Household income: men OR _low, middle _= 0.2-0.3, women = ns/Children aged 0-12 yo at home: men = ns, women = ns	Age, gender as a stratification variable, education, self-employment status, rotating shift, work hours, job strain.

Shields (2002) [130]	Workers aged 18- 54 years not working night shifts (*N *= 4,298). Canada.	Psychological distress (WHO-CIDI)	Up to 4	*Community* Occupational status: men b_sales/service _= 0.06, women = ns *Family* Marital status: men = ns, women = ns/Couple strains: men = ns, women = ns/Household income: men b_low _= -0.05, women = ns/Children aged 0-12 yo at home: men = ns, women = ns	Age, gender as a stratification variable, education, mastery, personal stress, smoking, alcohol consumption, physical activity, body mass index, mental health at baseline, self-employment status, week-end shifts, job strain, social support at work, physical demands, job insecurity, rotating shift, work hours.

Smith et al. (2008) [133]	Workers aged 25- 60 years working a minimum of 20 hours per week, not self-employed (*N *= 3,411). Canada.	Psychological distress (WHO-CIDI)	4	*Combined Community and Networks* Nb. of chronic stressors (friends, neighborhood): ns *Family* Nb. of chronic stressors (partner, child, parent): ns/Chronic exposure to financial stress: ns/Household income: ns	Age, gender, education, personal stress, self-rated health, body mass index, hypertension, heart disease, back pain, mental health at baseline, decision latitude.

Stansfeld et al. (1998) [134]	Civil servants aged 35-55 years at baseline (*N *= 8,315). United Kingdom.	Mental health functioning (SF-36)	Up to 8	*Combined Community and Networks* Social network index (friends, relatives; church, social clubs): men OR_Low, moderate _= 1.33-1.39, women = ns *Combined Networks and Family* Confiding support: men OR_low _= 1.60/Negative aspects of close relationships: men = ns, women OR _high, moderate _= 1.52-1.73. (closest nominated person in reference for all indicators)	Age, gender as a stratification variable, employment grade, negative affectivity, mental and physical health at baseline, decision latitude, job demands, social support at work, effort-reward imbalance.

Wickrama et al. (2005) [36]	Working parents (*N *= 692). United States.	Depression (SCL-90)	10	*Combined Networks and Family* Nb. of life events (partner, child, parent; friends): men B = 0.10, women B = 0.21	Gender as a stratification variable, education, mental health at baseline, decision latitude.

**Case-control**					
Ostry et al. (2006)^35 ^	Male sawmill workers (*N *= 822). Canada.	Neurotic disorder diagnosis (ICD9)	5	*Family* Marital status = ns	Duration of job, ethnicity, occupational status, job demands.

*Note*. OR: odds ratios; b: unstandardized betas; B: standardized betas; ns = non-significant association at *p *< 0.05.

### Nature and strength of the evidence linking non-work factors to mental health

As shown in Table 2 adequate methodological (*M *= 5.5, *SD *= 1.2) and conceptual (*M *= 4.3, *SD *= 1.4) quality described the analytical sample. The studies with the highest methodological quality scores [12,39,132] did not get the maximum score of 9 on the NOS scale due to non-representative samples of the general workforce[39,132], inclusion of workers with mental health problems at baseline[12,39], and ascertainment of mental health outcomes by a non-medical expert[12,132]. Overall, seven of the included studies were of relatively high methodological quality[12,35,39,40,130,132,133]. An examination of the analytical breadth of the selected studies revealed that all studies considered the family level of analysis, while a majority of them also extended their analyses to the networks [12,36-39,131,133,134] and/or the community or society (as per Table 1)[12,40,130-134]. The rest of this section discusses the strength of evidence for each analytical level of social organization and its indicators as reported in Table 3.

**Table 2:** Critical appraisal of the longitudinal cohort and case-control studies included for analysis

References	**Analytical breadth**^**a**^	**Analytical depth**^**a**^	**Selection**^**b**^	**Comparability**^**b**^	**Outcome/Exposure**^**b**^	**Total**^**c**^
**Cohorts**						
Barnett and Brennan (1998)[40]	**	***	**	**	**	5;6
Bromet et al. (1988)[39]	**	**	**	**	***	4;7
Fuhrer et al. (1999)[131]	***	***	*	**	*	6;4
Griffin et al. (2002)[37]	**	**	**	**	*	4;5
Marchand et al. (2005)[12]	***	***	***	**	**	6;7
Niedhammer et al. (1998)[38]	**	**	*	**	*	4;4
Revicki et al. (1993)[129]	*	*	*	**	*	2;4
Shields (1999)[132]	**	**	***	**	**	4;7
Shields (2002) [130]	**	***	**	**	**	5;6
Smith et al. (2008)[133]	***	**	**	**	**	5;6
Stansfeld et al. (1998)[134]	***	***	*	**	**	6;5
Wickrama et al. (2007)[36]	**	*	*	*	**	3;4
**Case-control**						
Ostry *et al*. (2006)[35]	*	*	**	**	**	2;6

*Note*. Full details on the scoring system are presented in Additional file 2.

^a ^Criterion considered for the conceptual assessment of the study quality.

^b ^Criterion considered for the methodological assessment of the study quality.

^c ^Total scores were obtained by summing the number of stars allocated to the conceptual and methodological components of the critical appraisal respectively.

**Table 3:** Summary of the strength of the evidence for non-work factors having an effect on workers' mental health

Analytical levels and indicators	Methodological quality	Consistency of **the findings**^**a**^	Nature of the **association**^**b**^	Strength of the evidence
**Family level**				
*Partner-specific indicators*				
Objective pathway	NOS≥6:[12,35,40,130,132]	NOS≥6:1/5 = 20% positive	Years in couple = + Marital status = .	Insufficient
	NOS < 6:[37,38,129,131]	NOS < 6:1/4 = 25% positive	Marital status = + +	
Subjective pathway	NOS≥6:[12,39,40,130]	NOS≥6:1/4 = 25% positive	Marital role quality = + Marital strains = .	Insufficient
	NOS < 6:[131,134]	NOS < 6:2/2 = 100% positive	Social support = +, + +	

*Child-specific indicators*				
Objective pathway	NOS≥6:[12,40,130,132]	NOS≥6:0/4 = 0% positive	Children at home = .	Insufficient
	NOS < 6:[37]	NOS < 6:0/1 = 0% positive	Children at home = .	
Subjective pathway	NOS≥6:[12]	NOS≥6:1/1 = 100% positive	Children strains = +	Insufficient

*Family structural characteristics*				
Objective pathway	NOS≥6:[12,40,130,132,133]	NOS≥6:0/5 = 0% positive	Family SES = -, --	Insufficient

*Global family stressors*				
Objective pathway	NOS≥6:[133]	NOS≥6:0/1 = 0% positive	Nb. chronic stressors = . Chronic financial stress = .	Insufficient
	NOS < 6: [36,38]	NOS < 6:2/2 = 100% positive	Nb. life events = +, + +	
Subjective pathway	NOS < 6:[37]	NOS < 6:1/1 = 100% positive	Home control = + +	Insufficient

**Network level**				
*Relative-specific indicators*				
Objective pathway	NOS < 6:[37]	NOS < 6:1/1 = 100% positive	Caregiving status = +	Insufficient

*Network structural characteristics*				
Objective pathway	NOS < 6:[131,134]	NOS < 6:1/2 = 50% positive	Network structure = +	Insufficient

*Global network stressors*				
Objective pathway	NOS≥6:[133]	NOS≥6:0/1 = 0% positive	Nb. chronic stressors = .	Insufficient
	NOS < 6: [36,38]	NOS < 6:2/2 = 100% positive	Nb. life events = +, + +	
Subjective pathway	NOS≥6:[12,39]	NOS≥6:1/2 = 50% positive	Social support = + +	Moderate
	NOS < 6:[131,134]	NOS < 6:2/2 = 100% positive	Social support = +, + +	

**Community/society level**				
*Community/society structural characteristics*				
Objective pathway	NOS≥6:[12,40,130,132]	NOS≥6:1/4 = 25% positive	Occupational structure = + Occupational SES = -	Insufficient
	NOS < 6:[131,134]	NOS < 6:1/2 = 50% positive	Community structure = +	

*Global community/society stressors*				
Objective pathway	NOS≥6:[133]	NOS≥6:0/1 = 0% positive	Nb. chronic stressors = .	Insufficient

^a ^A positive finding was considered if reported associations were significant at *p *< 0.05 and in the anticipated direction for exposure-outcome association.

^b^: non-significant association at *p *< 0.05; -: inverse association between non-work factors and mental health of modest magnitude (b, 2 > OR > 0.75); - -: inverse association between non-work factors and mental health of strong magnitude (OR 0.75≥ or ≥2); +: positive association of modest magnitude (b, 2 > OR > 0.75); + + : positive association between non-work factors and mental health of strong magnitude (OR 0.75≥ or ≥2).

#### Family

Partner relationships were systematically included in all studies. Consistent, non-significant evidence that marital status‚ as an objective indicator‚ affected mental health outcomes was reported by 4 high-[12,35,130,132] and 3 low-quality studies[37,129,131]. The one low-quality study that succeeded in modeling a significant, negative relationship between marital status and depressive symptoms in the GAZEL cohort investigated the effect of multiple modalities of relationships rather than the conventional dichotomy "alone/in relationship"[38]. Non-significant effects of subjectively measured maritally strained relationships were reported by 3 high-quality studies[12,39,130]. Comparatively two low-quality studies[131,134] from the Whitehall cohort found associations of modest magnitude for the subjective indicator of "partner's support". Of note, "partner's support" assessed perceived positive and negative aspects of social support from others whom respondents designated as "closest" to them, a designation that predominantly, though not universally, referred to partners. One high-quality study alternatively demonstrated a significant negative relationship between the objective indicator "years together" and the subjective indicator "marital-role quality" in relation with psychological distress in a sample of dual-earner couples[40]. The objective indicator for parental status yielded consistent, non-significant results (4 high-[12,40,130,132] and 1 low-quality studies[37]). One high-quality study based on the NPHS cohort found significant yet weak associations between child-related strains and psychological distress[12].

Other family-related indicators pertained to structural characteristics of the family and chronic or severe family-related stressors. Three high-quality studies[12,40,133] failed to reproduce a significant association between the objective indicator for household socioeconomic status and mental health outcomes, whereas one high-quality study[133] confirmed the absence of a similar effect for chronic financial stress. Two high-quality studies from the NPHS cohort reported a significant yet inverted effect of household income on major depressive disorders and psychological distress among men[130,132]. Two low-quality studies[36,38] found supportive evidence of an effect of life events, of which one from the GAZEL cohort reported results of modest magnitude for men and of high magnitude for women. In both studies, measurement of life events included items related to family members (e.g., partner, child, parent) and to the extended network (e.g., relatives, friends). By contrast, one high-quality study[133] did not find any association for family-specific life events (e.g., partner, child, parent) alone. Finally, one low-quality study showed modest to strong effects for the family stressor "home control" in the Whitehall cohort[37].

In summary, the evidence supporting an effect for family-level factors on workers' mental health appears to be insufficient. This conclusion holds regardless of the integration of indicators measuring the combined influence of family- and network-level into the analysis.

#### Networks

Eight studies examined the relationship between network features and workers' mental health. Of these, one low-quality study from the Whitehall cohort showed an association of modest magnitude between the objective indicator of providing care for an elderly or disabled relative and mental health outcomes among men[37]. From the same cohort, two low-quality studies [131,134] reported mixed evidence for network structural features (e.g., number of people in the network, frequency of contacts). As for network stressors, the conclusive results obtained by combining objectively measured network- and family-level life events, discussed above[36,38], were not reproduced when one high-quality study[133] jointly considered community-level events with network-level events. As for subjective measures, one high-quality study from the NPHS cohort reported strong protective effects for non-work social support[12], whereas two low-quality studies from the Whitehall cohort[131,134] noted modest to strong effects. These latter studies used broad expressions to describe network relationships (e.g., "nearest confidant", "someone" and "closest nominated persons"), whereas the only non-conclusive study used a group-specific indicator of social support ("friends")[39]. The evidence for effects on workers' mental health from network-level factors is therefore of moderate strength according to our scoring system but only for subjective indicators associated to social support.

#### Community/Society

In all, seven studies investigated the community/society analytical level. In terms of community/society structural characteristics, out of the four studies relying on national occupational classification systems to describe occupational status, prestige and average income[12,40,130,132], only one found an inverted protective association for lower socioeconomic occupational groups on psychological distress in the NPHS cohort[130]. Similarly, one high-quality study based on a multilevel analysis of the NPHS cohort showed a marginal but significant association between societal occupational structure and psychological distress after adjustment for individual-level factors[12]. Alternatively, two low-quality studies [131,134] reported inconsistent evidence of an association between a social network index based on network (e.g., relatives, friends) and community-member exchanges (e.g., visits to social clubs, church). One high-quality study[133] reported non-significant effects on psychological distress for joint network- and community-level life stressful events. No study assessed the relationship between community/society-level subjective indicators and workers' mental health. Support for an effect for community/society-level factors on workers' mental health has proven insufficient.

## Discussion

The aim of this systematic review was to provide robust conceptual and methodological guidelines for assessing the relative contribution of non-work determinants to workers' mental health above and beyond that of work determinants. In all, 13 longitudinal were evaluated for this review, among which 7 of these studies were of high methodological quality.

This review makes a salient contribution to the occupational stress literature by pointing out the lack of comprehensive and cumulative knowledge about the concomitant relationships between work and non-work domains in the explanation of workers' mental health. Indeed, among all potentially eligible longitudinal studies that met our selection criteria in terms of publication type, population, design, outcome and work exposure, the majority (*N *= 40/79; 50.6%) did not consider non-work factors, and nearly one third (*N *= 26/79; 32.9%) included non-work factors with no reports of their specific effect sizes. Moreover, when we examined the analytical breadth of the three levels of social organization considered (i.e., family, networks, community/society), we saw that the current state of knowledge about such concomitant relationships was essentially located at the family and network level. As far as analytical depth was concerned, although studies used multiple indicators of the non-work domain normatively, for a given non-work factor only a minority of studies sought to assess the joint contribution of objective and subjective measurement. Overall, we found insufficient evidence for any effects on workers' mental health of family-or community/society-level factors, although we did find evidence of moderate strength for social support at the network level.

These findings highlight important gaps in research on workers' mental health. Currently, mounting evidence shows that social features from every life environments are linked to mental health outcomes in the general population[135,136]. The nature of the pathways through which these life environments dynamically intersect with what goes on in the work environment raises critical issues with regard to the relational, spatial and temporal dynamics of workers' mental health. Unraveling such dynamics throughout the trajectory of workers' active life is also of significant interest for a wide range of public health-related issues such as work-life balance and civic participation[137,138]. This however can only be adequately addressed with the recognition that a greater attention ought to be paid to non-work determinants in the design of high quality longitudinal studies in the short term.

While highly informative, certain methodological limitations apply to this review. Firstly, we limited study eligibility to English- and French-language publications that did not refer to the grey literature. The strength of the confirmatory search strategy we developed‚ however‚ appeared exhaustive and comprehensive enough to eliminate significant omissions. The population we chose for analysis excluded studies based on clinical subjects due to potentially accrued individual vulnerability to stress. Further research is needed to thoroughly clarify this premise. Secondly, the heterogeneity of study design posed challenges for comparability. We partially addressed this limitation in our appraisal with the NOS instrument[139]. Hence, although the treatment of confounders was uneven, studies that minimally controlled for age and gender, which are considered primary determinants of mental health, received higher scores. Thirdly, we made full-workforce representativeness a reference criterion so that the dynamics we were investigating would remain generalizable. This threshold may appear high, but in our opinion it led to sounder conclusions concerning gender, age, and socioeconomic variations in the distribution of work, non-work factors, and mental health outcomes, which sampling strategies excluding any of these determinants might not have detected[35,39]. Fourthly, ascertaining outcomes using the NOS instrument was likely more consistent with epidemiological approaches to mental health outcomes. Alternative scoring for operationalizations based on a continuum however yielded the same results.

Lastly, the methodological decision to integrate studies with partial data overlap from a single cohort into our narrative synthesis merits additional consideration. This decision was initially informed by the need to translate a balance between the level of comprehensiveness necessitated to allow for such an exploratory synthesis to be conducted, and the level of restrictiveness in studies inclusion criteria necessitated to rigorously contain a conflation bias in the results. This was best achieved in our view by allowing multiple studies from a single cohort to be considered for evaluation following stringent criteria at different stages of our methodology (i.e., cumulative criteria for studies inclusion, high threshold for consistency in findings from the critical appraisal). As illustrated in Table 1, marginal overlaps in endpoints, work and non-work exposures and mental health outcomes were documented from the NPHS and the Whitehall studies. Again, we can tentatively hypothesize that distinctive causal dynamics potentially associated with design variations accounted for the substantive differences observed in results for comparable indicators between studies from a single cohort. A critical reflection as to the extent to which overlap in causal dynamics in studies from single cohorts should be validly considered in future systematic reviews is warranted.

### Research implications

This systematic review identified two key recommendations that should be of immediate interests for research on workers' mental health.

#### Recommendation 1

We recommend that future longitudinal research systematically consider both work and non-work determinants of workers' mental health. In this review, 9 out of 13 studies were successful in detecting significant and independent effects over time on outcomes for non-work factors after controlling for work factors and other individual-level characteristics such as age, gender, lifestyles and past mental health history. Yet, lack of cumulative knowledge rather than inconsistency in results emerged as the primary reason that the evidence for effects of the non-work factors on workers' mental health was only modest. All analytical levels (i.e., family, networks, community/society) and their respective indicators (i.e., subjective, objective) should be prioritized.

#### Recommendation 2

We further recommend that robust methodological and conceptual parameters be explicitly stated and applied. Careful considerations about the conceptualization and operationalization of the non-work domain are warranted given that its construct definition captures distinct levels of social organization. The opportunity to analytically and empirically untangle in a straightforward way the specific effects of work and non-work indicators is paramount should evidence-based interventions and policy be efficiently informed by longitudinal studies targeting workers' mental health.

## Conclusion

By combining insights of several disciplines such as epidemiology and sociology, this systematic review has outlined that the non-work domain is a largely underinvestigated area of research pertaining to the study of workers' mental health. In the future, it is only by rigorously addressing the quality of the state of the knowledge both from a conceptual and methodological standpoint that healthy workplace policy, intervention and research can comprehensively balance the ways in which work and non-work domains jointly contribute to the explanation of workers' mental health.

## Competing interests

The authors declare that they have no competing interests.

## Authors' contributions

NB planned, collected, and analysed the data, and is lead author. AM and MEB assisted in the conceptual and verification stages of the study. All authors read and approved the final manuscript. Ethics approval was not required for this systematic review.

## Pre-publication history

The pre-publication history for this paper can be accessed here:

http://www.biomedcentral.com/1471-2458/11/439/prepub

## Supplementary Material

Additional file 1 **Search Strategy**. It contains all the details for the search strategy performed for the research article. Click here for file

Additional file 2 **Critical Appraisal**. It contains a table entitled 'Additional file 2. Items considered for the critical appraisal'. This table includes all the items upon which the methodological and conceptual quality of the included studies for the systematic review were critically appraised. Click here for file

Additional file 3 **Strength of Evidence**. It contains a figure entitled 'Additional file 3. Strength of evidence assessment'. This figure illustrates the decision process followed in the assessment of the strength of evidence. Click here for file

Additional file 4 **Moose**. It contains a table entitled 'Additional file 4. MOOSE Checklist'. This table includes all items upon which an evaluation of the research article was based considering the MOOSE evaluation tool. Click here for file
