# Supplementary material for: What do we know about the non-work determinants of workers' mental health? A systematic review of longitudinal studies
Source: BMC Public Health. 2011 Jun 6;11:439. doi: 10.1186/1471-2458-11-439 (PMC3141446; doi:10.1186/1471-2458-11-439)
Supplement: Additional file 1 — Search Strategy. It contains all the details for the search strategy performed for the research article. [file 1471-2458-11-439-S1.DOC]

**Additional file 1. Search strategy**

**1. OVID**

- - Cinhal

1. exp Family/

2. ((strain$ or stress$ or conflict$) adj25 (marital or marriage or spouse)).mp.

3. ((strain$ or stress$ or conflict$) adj25 (parent$ or child$ or family)).mp.

4. (work$ adj25 (home or family)).mp.

5. exp social networks/

6. (network$ adj social).mp.

7. (network$ adj25 (ties or size or range or density or dense or bound$ or proxim$ or homogene$ or reachab$)).mp.

8. exp social support/

9. (social adj support).mp.

10. exp residence characteristics/

11. exp communities/

12. exp social environment/

13. exp work environment/

14. 12 not 13

15. (neighborhood$ or (health adj3 place)).mp. [mp=title, subject heading word, abstract, instrumentation]

16. exp social capital/

17. (social adj capital).mp.

18. ((social adj cohesion) or violence or crime).mp.

19. (social adj engagement).mp.

20. (deprivation or disadvantage or inequalit$ or poverty).mp.

21. (social adj participation).mp.

22. (collective adj efficacy).mp.

23. exp social change/ or exp socioeconomic factors/ or exp CULTURE/ or exp Politics/

24. ((societ$ or communit$ or neighbor$) adj25 (cultur$ or $economic$ or politic$ or change)).mp.

25. ((social or psychosocial) adj3 (environ$ or context$)).mp.

26. ((social or psychosocial) adj (stress$ or strain$)).mp.

27. exp ENVIRONMENTAL POLLUTION/

28. exp occupational exposure/

29. 27 not 28

30. (physical adj3 (environ$ or context$)).mp.

31. ((public or community) adj3 (service$ or facilit$ or amenit$ or infrastructure$)).mp. [mp=title, subject heading word, abstract, instrumentation]

32. or/1-31

33. exp Work Load/

34. exp Occupational stress/

35. (Karasek* or Siegrist*).mp.

36. ((demand$ adj3 control model) or (demand$ adj3 support model)).mp.

37. ((effort adj reward) or (effort adj3 imbalance)).mp.

38. (social adj support adj500 (colleague$ or superior$ or supervisor$)).mp.

39. (((skill$ or competenc$) adj discretion) or ((skill$ or competenc$) adj utilization) or ((work or job) adj control) or (decision adj (latitude or authority or autonomy)) or (autonomy adj (work or job))).mp.

40. ((psychological or job or work or mental) adj3 (demand$ or load)).mp.

41. ((job or work or employ$) adj insecurity).mp.

42. ((organizational or occupational or procedural or distributive) adj $justice).mp.

43. (physical adj (demand$ or load)).mp.

44. ((work$ or job or occupation$ or organization$) adj psychosocial).mp.

45. ((work$ or occupation$ or job or organization$) adj (strain$ or stress$)).mp.

46. (work$ adj conflict$).mp.

47. (work$ adj (schedul$ or hour$)).mp.

48. or/33-47

49. exp psychological stress/

50. exp occupational stress/

51. 49 not 50

52. exp Burnout, Professional/

53. DEPRESSION/ or DEPRESSION, REACTIVE/

54. exp Adjustment Disorders/

55. exp Mood Disorders/

56. exp Neurotic Disorders/

57. burnout.mp.

58. ((depressive adj2 disorder$) or (affective adj2 disorder$) or (mood adj2 disorder$) or (neurotic adj2 disorder$) or depression).mp.

59. (psychol$ adj3 distress).mp.

60. (psychol$ adj3 stress).mp.

61. (GHQ or General Health questionnaire or Kessler Psychological Distress or Ilfeld Psychiatric Symtoms or PERI Life Events or General Well-Being Schedule or Affect Balance Scale or Composite International Diagnostic Interview or Center of Epidemiologic Studies Depression or (Hospital Anxiety and Depression) or Rutter Malaise Inventory).mp.

62. or/49-61

63. exp Prospective Studies/

64. exp Retrospective Design/

65. (causal$ or prognos* or predict* or course* or risk*).mp.

66. Prognosis/

67. INCIDENCE/

68. Risk Factors/

69. ((longitudinal or prospectiv$ or retrospectiv$ or cohort or follow up) and (stud$ or design$)).mp.

70. or/63-69

71. 32 and 48 and 62 and 70

72. limit 71 to (english or french)

73. letter.pt.

74. editorial.pt.

75. 72 not (73 or 74)

- - EMBASE

1. exp Family/

2. ((strain$ or stress$ or conflict$) adj25 (marital or marriage or spouse)).mp.

3. ((strain$ or stress$ or conflict$) adj25 (parent$ or child$ or family)).mp.

4. (work$ adj25 (home or family)).mp.

5. exp social network/

6. (network$ adj social).mp.

7. (network$ adj25 (ties or size or range or density or dense or bound$ or proxim$ or homogene$ or reachab$)).mp.

8. exp social support/

9. (social adj support).mp.

10. exp community/

11. exp social environment/ not (exp public opinion/ or exp organizational climate/)

12. (neighborhood$ or (health adj3 place)).mp. [mp=title, abstract, subject headings, heading word, drug trade name, original title, device manufacturer, drug manufacturer name]

13. (social adj capital).mp.

14. ((social adj cohesion) or violence or crime).mp.

15. (social adj engagement).mp.

16. (deprivation or disadvantage or inequalit$ or poverty).mp.

17. (social adj participation).mp.

18. (collective adj efficacy).mp.

19. exp social change/ or exp socioeconomics/ or exp Politics/

20. ((societ$ or communit$ or neighbor$) adj25 (cultur$ or $economic$ or politic$ or change)).mp.

21. ((social or psychosocial) adj3 (environ$ or context$)).mp.

22. ((social or psychosocial) adj (stress$ or strain$)).mp.

23. exp ENVIRONMENTAL POLLUTION/

24. (physical adj3 (environ$ or context$)).mp.

25. ((public or community) adj3 (service$ or facilit$ or amenit$ or infrastructure$)).mp.

26. or/1-25

27. exp Work Load/

28. exp Occupational stress/

29. (Karasek* or Siegrist*).mp.

30. ((demand$ adj3 control model) or (demand$ adj3 support model)).mp.

31. ((effort adj reward) or (effort adj3 imbalance)).mp.

32. (social adj support adj500 (colleague$ or superior$ or supervisor$)).mp.

33. (((skill$ or competenc$) adj discretion) or ((skill$ or competenc$) adj utilization) or ((work or job) adj control) or (decision adj (latitude or authority or autonomy)) or (autonomy adj (work or job))).mp.

34. ((psychological or job or work or mental) adj3 (demand$ or load)).mp.

35. ((job or work or employ$) adj insecurity).mp.

36. ((organizational or occupational or procedural or distributive) adj $justice).mp.

37. (physical adj (demand$ or load)).mp.

38. ((work$ or job or occupation$ or organization$) adj psychosocial).mp.

39. ((work$ or occupation$ or job or organization$) adj (strain$ or stress$)).mp.

40. (work$ adj conflict$).mp.

41. (work$ adj (schedul$ or hour$)).mp.

42. or/27-41

43. exp psychological stress/

44. exp Burnout, Professional/

45. exp DEPRESSION/

46. exp Adjustment Disorders/

47. exp Mood Disorders/

48. exp Neurotic Disorders/

49. burnout.mp.

50. ((depressive adj2 disorder$) or (affective adj2 disorder$) or (mood adj2 disorder$) or (neurotic adj2 disorder$) or depression).mp.

51. (psychol$ adj3 distress).mp.

52. (psychol$ adj3 stress).mp.

53. (GHQ or General Health questionnaire or Kessler Psychological Distress or Ilfeld Psychiatric Symtoms or PERI Life Events or General Well-Being Schedule or Affect Balance Scale or Composite International Diagnostic Interview or Center of Epidemiologic Studies Depression or (Hospital Anxiety and Depression) or Rutter Malaise Inventory).mp.

54. or/43-53

55. exp Prospective Study/ or exp Retrospective study/ or exp Longitudinal study/

56. (causal$ or prognos* or predict* or course* or risk*).mp.

57. Prognosis/

58. INCIDENCE/

59. Risk Factors/

60. ((longitudinal or prospectiv$ or retrospectiv$ or cohort or follow up) and (stud$ or design$)).mp.

61. or/55-60

62. 26 and 42 and 54 and 61

63. limit 62 to human

64. limit 63 to (english or french)

65. letter.pt.

66. editorial.pt.

67. 64 not (65 or 66)

- - PsycINFO

1. exp Family/

2. ((strain$ or stress$ or conflict$) adj25 (marital or marriage or spouse)).mp.

3. ((strain$ or stress$ or conflict$) adj25 (parent$ or child$ or family)).mp.

4. (work$ adj25 (home or family)).mp.

5. exp social network/

6. (network$ adj social).mp.

7. (network$ adj25 (ties or size or range or density or dense or bound$ or proxim$ or homogene$ or reachab$)).mp.

8. exp social support/

9. (social adj support).mp.

10. exp social environment/ not (exp academic environment/ or exp animal environment/ or working conditions/)

11. (neighborhood$ or (health adj3 place)).mp. [mp=title, abstract, heading word, table of contents, key concepts]

12. exp social capital/

13. (social adj capital).mp.

14. ((social adj cohesion) or violence or crime).mp.

15. (social adj engagement).mp.

16. (deprivation or disadvantage or inequalit$ or poverty).mp.

17. (social adj participation).mp.

18. (collective adj efficacy).mp.

19. exp social change/ or exp culture/ or exp socioeconomics/ or exp Politics/

20. ((societ$ or communit$ or neighbor$) adj25 (cultur$ or $economic$ or politic$ or change)).mp.

21. ((social or psychosocial) adj3 (environ$ or context$)).mp.

22. ((social or psychosocial) adj (stress$ or strain$)).mp.

23. exp pollution/

24. (physical adj3 (environ$ or context$)).mp.

25. ((public or community) adj3 (service$ or facilit$ or amenit$ or infrastructure$)).mp.

26. or/1-25

27. exp Work Load/

28. exp Occupational stress/ not burnout.mp.

29. (Karasek* or Siegrist*).mp.

30. ((demand$ adj3 control model) or (demand$ adj3 support model)).mp.

31. ((effort adj reward) or (effort adj3 imbalance)).mp.

32. (social adj support adj500 (colleague$ or superior$ or supervisor$)).mp.

33. (((skill$ or competenc$) adj discretion) or ((skill$ or competenc$) adj utilization) or ((work or job) adj control) or (decision adj (latitude or authority or autonomy)) or (autonomy adj (work or job))).mp.

34. ((psychological or job or work or mental) adj3 (demand$ or load)).mp.

35. ((job or work or employ$) adj insecurity).mp.

36. ((organizational or occupational or procedural or distributive) adj $justice).mp.

37. (physical adj (demand$ or load)).mp.

38. ((work$ or job or occupation$ or organization$) adj psychosocial).mp.

39. ((work$ or occupation$ or job or organization$) adj (strain$ or stress$)).mp.

40. (work$ adj conflict$).mp.

41. (work$ adj (schedul$ or hour$)).mp.

42. or/27-41

43. exp psychological stress/

44. exp DEPRESSION/

45. exp Adjustment Disorders/

46. exp Mood Disorders/

47. burnout.mp.

48. burnout.mp.

49. ((depressive adj2 disorder$) or (affective adj2 disorder$) or (mood adj2 disorder$) or (neurotic adj2 disorder$) or depression).mp.

50. (psychol$ adj3 distress).mp.

51. (psychol$ adj3 stress).mp.

52. (GHQ or General Health questionnaire or Kessler Psychological Distress or Ilfeld Psychiatric Symtoms or PERI Life Events or General Well-Being Schedule or Affect Balance Scale or Composite International Diagnostic Interview or Center of Epidemiologic Studies Depression or (Hospital Anxiety and Depression) or Rutter Malaise Inventory).mp.

53. or/43-52

54. exp Prospective Studies/ or exp Retrospective studies/ or exp Longitudinal studies/ or exp follow up studies/

55. (causal$ or prognos* or predict* or course* or risk*).mp.

56. Prognosis/

57. INCIDENCE/

58. Risk Factors/

59. ((longitudinal or prospectiv$ or retrospectiv$ or cohort or follow up) and (stud$ or design$)).mp.

60. or/54-59

61. 26 and 42 and 53 and 60

62. limit 61 to human

63. limit 62 to (english or french)

64. limit 63 to (editorial or letter)

65. 63 not 64

- - MEDLINE

1. exp Family/

2. ((strain$ or stress$ or conflict$) adj25 (marital or marriage or spouse)).mp.

3. ((strain$ or stress$ or conflict$) adj25 (parent$ or child$ or family)).mp.

4. (work$ adj25 (home or family)).mp.

5. exp social network/

6. (network$ adj social).mp.

7. (network$ adj25 (ties or size or range or density or dense or bound$ or proxim$ or homogene$ or reachab$)).mp.

8. exp social support/

9. (social adj support).mp.

10. exp community/

11. exp social environment/

12. (neighborhood$ or (health adj3 place)).mp. [mp=title, original title, abstract, name of substance word, subject heading word]

13. (social adj capital).mp.

14. ((social adj cohesion) or violence or crime).mp.

15. (social adj engagement).mp.

16. (deprivation or disadvantage or inequalit$ or poverty).mp.

17. (social adj participation).mp.

18. (collective adj efficacy).mp.

19. exp social change/ or exp socioeconomics/ or exp Politics/

20. ((societ$ or communit$ or neighbor$) adj25 (cultur$ or $economic$ or politic$ or change)).mp.

21. ((social or psychosocial) adj3 (environ$ or context$)).mp.

22. ((social or psychosocial) adj (stress$ or strain$)).mp.

23. exp ENVIRONMENTAL POLLUTION/

24. (physical adj3 (environ$ or context$)).mp.

25. ((public or community) adj3 (service$ or facilit$ or amenit$ or infrastructure$)).mp.

27. exp Work Load/

28. exp Occupational stress/

29. (Karasek* or Siegrist*).mp.

30. ((demand$ adj3 control model) or (demand$ adj3 support model)).mp.

31. ((effort adj reward) or (effort adj3 imbalance)).mp.

32. (social adj support adj500 (colleague$ or superior$ or supervisor$)).mp.

33. (((skill$ or competenc$) adj discretion) or ((skill$ or competenc$) adj utilization) or ((work or job) adj control) or (decision adj (latitude or authority or autonomy)) or (autonomy adj (work or job))).mp.

34. ((psychological or job or work or mental) adj3 (demand$ or load)).mp.

35. ((job or work or employ$) adj insecurity).mp.

36. ((organizational or occupational or procedural or distributive) adj $justice).mp.

37. (physical adj (demand$ or load)).mp.

38. ((work$ or job or occupation$ or organization$) adj psychosocial).mp.

39. ((work$ or occupation$ or job or organization$) adj (strain$ or stress$)).mp.

40. (work$ adj conflict$).mp.

41. (work$ adj (schedul$ or hour$)).mp.

42. or/27-40

43. exp psychological stress/

44. exp Burnout, Professional/

45. exp DEPRESSION/

46. exp Adjustment Disorders/

47. exp Mood Disorders/

48. exp Neurotic Disorders/

49. burnout.mp.

50. ((depressive adj2 disorder$) or (affective adj2 disorder$) or (mood adj2 disorder$) or (neurotic adj2 disorder$) or depression).mp.

51. (psychol$ adj3 distress).mp.

52. (psychol$ adj3 stress).mp.

53. (GHQ or General Health questionnaire or Kessler Psychological Distress or Ilfeld Psychiatric Symtoms or PERI Life Events or General Well-Being Schedule or Affect Balance Scale or Composite International Diagnostic Interview or Center of Epidemiologic Studies Depression or (Hospital Anxiety and Depression) or Rutter Malaise Inventory).mp.

54. or/43-53

55. exp Prospective Study/ or exp Retrospective study/ or exp Longitudinal study/

56. (causal$ or prognos* or predict* or course* or risk*).mp.

57. Prognosis/

58. INCIDENCE/

59. Risk Factors/

60. ((longitudinal or prospectiv$ or retrospectiv$ or cohort or follow up) and (stud$ or design$)).mp.

61. or/55-60

62. 26 and 42 and 54 and 61

63. limit 62 to human

64. limit 63 to (english or french)

65. letter.pt.

66. editorial.pt.

67. 64 not (65 or 66)

- - EBM Reviews (idem Medline)

**2. ISI Web of Science**

- - Science Citation Index Expanded (SCI-EXPANDED)
  - Social Sciences Citation Index (SSCI)
  - Arts & Humanities Citation Index (A&HCI)

| #20 | ts= ((ENVIRONMENT* POLLUTION ) or (physical and (environ* or context*))) |
| --- | --- |
| #19 | ts=((social or psychosocial) and (stress* or strain*)) |
| #18 | ts= ((social or psychosocial) and (environ* or context*)) |
| #17 | ts= ((communit* or societ*) and (social capital or social cohesion or social engagement or deprivation or disadvantage or inequalit* or poverty or social participation or collective efficacy or social change or socioeconomic* or Politics)) |
| #16 | ts=(social network* or (network* and (size or range or density or dense or bound* or proxim* or homogene* or reachab*)) or social support) |
| #15 | ts= (work-home or work-family or spillover) |
| #14 | #13 OR #12 OR #11 OR #10 OR #9 OR #8 OR #7 OR #6 OR #5 OR #4 OR #3 OR #2 OR #1 |
| #13 | ts= (work* and (schedul* or hour*)) |
| #12 | ts=(work conflict*) |
| #11 | ts=((work* or occupation* or job or organization*) and (strain* or stress*)) |
| #10 | ts=((work* or job or occupation* or organization*) and psychosocial) |
| #9 | ts=(physical and (demand* or load)) |
| #8 | ts=((organizational or occupational or procedural or distributive) and justice) |
| #7 | ts=((job or work or employ*) and security) |
| #6 | ts=((psychological or job or work or mental) and (demand* or load)) |
| #5 | ts=(((skill$* or competenc*) and discretion) or ((skill* or competenc*) and utilization) or ((work or job) and control) or (decision and (latitude or authority or autonomy)) or (autonomy adj (work or job))) |
| #4 | ts=((social support and work) or (social support and colleagues) or (social support and supervisor*) or (social support and manager*) or (social support and superior*)) |
| #3 | ts=((effort reward) or (effort imbalance)) |
| #2 | ts=((demand* control model) or (demand* support model)) |
| #1 | ts=(Karasek* or Siegrist*) |
