# Supplementary material for: What do we know about the non-work determinants of workers' mental health? A systematic review of longitudinal studies
Source: BMC Public Health. 2011 Jun 6;11:439. doi: 10.1186/1471-2458-11-439 (PMC3141446; doi:10.1186/1471-2458-11-439)
Supplement: Additional file 2 — Critical Appraisal. It contains a table entitled 'Additional file 2. Items considered for the critical appraisal'. This table includes all the items upon which the methodological and conceptual quality of the included studies for the systematic review were critically appraised. [file 1471-2458-11-439-S2.DOC]

**Additional file 2**. Items considered for the critical appraisal

| Criteria | Design |
| --- | --- |
| **Methodological evaluation** |  |
| *Selection* |  |
| Exposed cohort representative of the overall occupational structure | CH |
| Non-exposed cohort drawn from the same community as the exposed cohort | CH |
| Ascertainment of exposure based on objective evaluations or structured interviews | CH |
| Outcome absent at start of study | CH |
| Case definition resulted from an independent validation (i.e., primary health record) | CC |
| Cases representative of the overall occupational structure | CC |
| Controls drawn from the same community as the cases | CC |
| Cases and controls with no history of mental health | CC |
| *Comparability* |  |
| Study controlled for age | CH, CC |
| Study controlled for gender | CH, CC |
| *Outcome/Exposure* |  |
| Outcome ascertained through medical expert diagnosis | CH |
| Exposure ascertained through structured interviews blinded to case/control status | CC |
| Outcome-exposure association evaluated for a minimum of 12 months | CH, CC |
| Follow-up participation rate was ≥80%  Non-response rate for cases and controls identical | CH  CC |
| **Conceptual evaluation** |  |
| *Analytical breadth* |  |
| Positive if data on non-work factors a) were included in multivariate analyses with work factors, and b) reported size effects.  *: One analytical level considered  **: Two analytical levels considered  ***: Three or more analytical levels considered | CH, CC |
| *Analytical depth* |  |
| Positive if data on non-work factors: a) were included in multivariate analyses with work factors, and b) reported size effects.  *: One indicator of non-work factor  **: Two indicators of non-work factors or more  ***: Two indicators of non-work factors or more, assessing both objective and subjective pathways of a given construct | CH, CC |

*Note*. Items for the methodological component of the critical evaluation were derived from the New Castle-Ottawa scale. A star was allocated based on a positive answer to the above-mentioned criteria. CC: case-control study; CH: cohort study.
