# Supplementary material for: What do we know about the non-work determinants of workers' mental health? A systematic review of longitudinal studies
Source: BMC Public Health. 2011 Jun 6;11:439. doi: 10.1186/1471-2458-11-439 (PMC3141446; doi:10.1186/1471-2458-11-439)
Supplement: Additional file 3 — Strength of Evidence. It contains a figure entitled 'Additional file 3. Strength of evidence assessment'. This figure illustrates the decision process followed in the assessment of the strength of evidence. [file 1471-2458-11-439-S3.DOC]

Yes

Yes

Q1. Is there 1 study with NOS score > mean NOS score?

No

**Strength of evidence**

**Criteria**

Yes

Q2. Consistency of the findings at 75% from multiple studies of which one is of high-quality?

No

Q3. Strong magnitude established at OR 0.75≥ or ≥2?

No

Moderate

**Scenarios**

High

1. A (+), B (-) or A (+), e (-)

2. A (+), B (+), C (-) or A (+), B (+), e (-)

3. A (+), B (+), C (-), D(-)

4. A (+), e (-), f (-), g (-)

*Rule.* Consistency lesser than 75% between high-quality studies or a mixture of high- and low-quality studies (scenarios 1-3), or consistency found only among low-quality studies (scenario 4).

1. A (++), B (++), C (+)

2. A (+), e (++), f (+), g (-)

3. A (++), e (++)

*Rule.* Consistency reached at 75% in the anticipated direction for exposure-outcome association independent of strength of magnitude (scenarios 1-2; Q3), or strong magnitude is evident from a mixture of high- and low-quality studies (scenario 3, Q4).

1. e (+), f (+)

*Rule*. Strength of evidence insufficient since no relatively high-quality study can corroborate findings from relatively low-quality studies.

1. A (++), B (++)

2. A (++), B (++), C (++), D (++/+/-/--)

3. A (++), B (++), C (++), e (++/+/-/--)

*Rule.* Cumulative positive answers for all criteria.

Insufficient

Q4. Do all studies have NOS score > mean NOS score?

Yes

Insufficient

**Additional file 3. Decision process for strength of evidence assessment**

**Additional file 3. Strength of evidence assessment**
