# Supplementary material for: What do we know about the non-work determinants of workers' mental health? A systematic review of longitudinal studies
Source: BMC Public Health. 2011 Jun 6;11:439. doi: 10.1186/1471-2458-11-439 (PMC3141446; doi:10.1186/1471-2458-11-439)
Supplement: Additional file 4 — Moose. It contains a table entitled 'Additional file 4. MOOSE Checklist'. This table includes all items upon which an evaluation of the research article was based considering the MOOSE evaluation tool. [file 1471-2458-11-439-S4.DOC]

**Additional file 4.** MOOSE Checklist

| Criteria | | | Description of the application of the criteria in the systematic review |
| --- | --- | --- | --- |
| **Reporting of background should include** | | |  |
| √ | Problem definition | | Little is known about the relative contribution of non-work determinants in the prediction of workers' mental health above and beyond that of work determinants. |
| √ | Hypothesis statement | | Non-work determinants are both important to causally explain workers' mental health still, knowledge about them is sparse and highly heterogeneous. |
| √ | Description of study outcomes | | Psychological distress, burnout, depression |
| √ | Type of exposure or intervention used | | Work determinants (psychosocial work environment, work contract) and non-work determinants (family, networks, community/society) |
| √ | Type of study designs used | | Longitudinal observational studies (case-control studies, cohort studies) |
| √ | Study population | | Community-based samples of workers |
| **Reporting of search strategy should include** | | |  |
| √ | Qualifications of searchers | | Affiliation and status for all authors provided in the cover letter |
| √ | Search strategy, including time period included in the synthesis and keywords | | Based on: 1) use of indexed and free terms; 2) full disclosure of the search strategy in Additional file 1; 3) use of a confirmatory search strategy without filters related to the non-work domain to insure comprehensiveness of the data. |
| √ | Databases and registries searched | | Cinhal (Ovid), Psycinfo (Ovid), Embase (Ovid), Medline (Ovid), EBM (all databases, Ovid), Sociological Abstracts (ISI Web), the Social Sciences Citation Index (ISI Web), and the Arts and Human Citation Index (ISI Web) |
| √ | Search software used, name and version, including special features | | Nvivo 2.0 for the storage and coding of abstracts. SPSS 15.0 for the descriptive statistics and storage of data. |
| √ | Use of hand searching | | Bibliographies of included papers were hand-searched for additional references |
| √ | List of citations located and those excluded, including justifications | | Flow chart from Figure 1 details the inclusion/exclusion decisional process followed, citation list available upon request. |
| √ | Method of addressing articles published in languages other than English | | We clarified that articles searched were in French and English. |
| √ | Method of handling abstracts and unpublished studies | | We clarified that the grey literature was excluded from the analysis. |
| √ | Description of any contact with authors | | We clarified that no author was contacted given that the data provided sufficient information. |
| **Reporting of methods should include** | | |  |
| √ | Description of relevance or appropriateness of studies assembled for assessing the hypothesis to be tested | | Detailed inclusion and exclusion criteria were described in the Methods section. |
| √ | Rationale for the selection and coding of data | | Data extracted from each of the studies were relevant to the population characteristics, study design, exposure, outcome. Scoring system is found in Additional file 2. |
| √ | Assessment of confounding | | We excluded from our study potential confounders related to limited duration, and clinical-based samples. |
| √ | Assessment of study quality, including blinding of quality assessors | | Use of the validated instrument NewCastle-Ottawa scale for the methodological quality assessment, creation of a specific instrument for the conceptual assessment with explicit details about the scoring system. Multiple independent interraters assessment and related statistics provided. |
| √ | Assessment of heterogeneity | | Not applicable |
| √ | Description of statistical methods in sufficient detail to be replicated | | Tables provide all necessary information for all results to be independently replicated. |
| √ | Provision of appropriate tables and graphics | | Figure 1 is a flow chart, Table 1 describes features of included studies, Table 2 provides scores from critical appraisals, Table 3 provides details from the evaluation of the strength of the evidence, Additional files 2 and 3 detail the critical assessment process applied. |
| **Reporting of results should include** | | |  |
| √ | Graph summarizing individual study estimates and overall estimate | | Not applicable |
| √ | Table giving descriptive information for each study included | | Table 1 describes the design, type of outcome and exposure, length of follow-up, and adjustments. |
| √ | Results of sensitivity testing | | Not applicable |
| √ | Indication of statistical uncertainty of findings | | Not applicable |
| **Reporting of discussion should include** | | |  |
| √ | Quantitative assessment of bias | | Not applicable |
| √ | Justification for exclusion | | Clinical-based samples were excluded on the grounds that increased individual vulnerability to stress and increase the level of heterogeneity in results. |
| √ | Assessment of quality of included studies | | We used the NewCastle-Ottawa scale for the methodological component of our critical appraisal, and the Additional file 2 provides full details of the criteria considered for its conceptual component. |
| **Reporting of conclusions should include** | | |  |
| √ | | Consideration of alternative explanations for observed results | We discussed the impact of our methodological choices on the results, emphasizing the comprehensiveness of the search strategy and stability of the results in the light of alternative methodological reasoning. |
| √ | | Generalization of the conclusions | We discussed that our results are representative of 13 out of 79 (16.4%) longitudinal studies in the field of workers' mental health matching selection criteria. Other studies predominantly fail to address non-work determinants (n=40/79; 50.6%) or merely adjusted for them with no reports of their estimated effect sizes (n=26/79; 32.9%). |
| √ | | Guidelines for future research | We recommend future studies on the inclusion of both non-work and work determinants of workers' mental health to increase cumulative knowledge lacking at the moment. |
| √ | | Disclosure of funding source | Not applicable |
